# Student Health Implications of School Closures during the COVID-19 Pandemic: New Evidence on the Association of e-Learning, Outdoor Exercise, and Myopia

**DOI:** 10.3390/healthcare9050500

**Published:** 2021-04-23

**Authors:** Ji Liu, Baihuiyu Li, Qiaoyi Chen, Jingxia Dang

**Affiliations:** 1 Faculty of Education, Shaanxi Normal University, Xi’an 710062, China; jiliu@snnu.edu.cn (J.L.); lilyli000627@outlook.com (B.L.); 2 School of Basic Medical Sciences, Xi’an Jiaotong University, Xi’an 710061, China; 3 The First Affiliated Hospital, Xi’an Jiaotong University, Xi’an 710061, China

**Keywords:** e-learning, youth and children health, visual health, myopia, COVID-19

## Abstract

The coronavirus disease 2019 (COVID-19) pandemic forced many education systems to consider alternative remote e-learning modalities, which have consequential behavioral and health implications for youth. In particular, increased e-learning engagement with digital screens and reduction in outdoor activities are two likely channels posing adverse risks for myopia development. This study investigated the association between e-learning screen use, outdoor activity, lighting condition, and myopia development among school-age children in China, during the first wave of the COVID-19 pandemic. Data were collected from 3405 school-age children attending primary, lower-secondary, and upper-secondary schools in China. Univariate parametric and nonparametric tests, and multivariate logistic regression analysis were used. Findings show that each diopter hour increase in daily e-learning screen use is significantly associated with progression of myopia symptoms (OR: 1.074, 95% CI: 1.058–1.089; *p* < 0.001), whereas engaging in outdoor exercise four to six times per week (OR: 0.745, 95% CI: 0.568–0.977; *p* = 0.034) and one to three times per week (OR: 0.829, 95% CI: 0.686–0.991; *p* = 0.048) is associated with a lower likelihood of myopia progression than none at all. In addition, we found that indoor lighting that is either “too dim” (OR: 1.686, 95% CI: 1.226–2.319; *p* = 0.001) or “too bright” (OR: 1.529, 95% CI: 1.007–2.366; *p* = 0.036) is significantly associated higher likelihood of myopic symptoms. Findings in this study uncover the less observable vision consequences of the COVID-19 pandemic on youths through digital online learning and highlight the importance of considering appropriate mitigation strategies to deal with this emerging public health challenge.

## 1. Introduction

The outbreak and spread of the coronavirus disease 2019 (COVID-19) pandemic have greatly impacted education systems worldwide, with more than 190 countries/territories closing schools partially or in full during peak months, affecting at least 1.5 billion school-age children [1]. In order to minimize learning disruptions and to resume proper functioning of educational activities, more than 60 educational systems have elected to partially re-open schools by offering online remote instruction and supplementary digital learning modalities [2]. While replacing print textbooks with e-learning arrangements and swapping in-person classroom teaching with online video conferencing provides reasonable and timely solutions to deal with challenges of pandemic-led school closures, the adverse vision consequences associated with these ad hoc emergency and crisis arrangements may be substantial [3], especially considering new eye-use routines during the pandemic and its consequent behavioral implications for young children whose sensory function is going through critical development [4]. Notably, the current global increase in myopia, which is a major factor contributing to irreversible blindness, has become a leading public health concern. By 2050, it is projected that 50% of the world population will be affected [5], which underscores the need to investigate the relevant risk factors, as well as potential mitigation strategies to combat this public health challenge.

In this regard, it is hypothesized that the reduction of outdoor activity due to school closures and home confinement, coupled with the overlapping increase in e-learning digital screen use as a result of remote e-learning arrangements, presents critical vision development risk factors that could propel higher myopia incidence and progression by re-shaping daily physical and learning behaviors. On the one hand, intensive use of digital screens for extended periods of time can have detrimental effects on children’s vision health development. Particularly, since eye development occurs throughout early stages of life, in vivo studies have suggested that prolonged near-vision stimulation can result in premature hyperopic defocus, which triggers compensating axial myopic eye growth and refractive vision development [6]. On the other hand, reduction in outdoor activities due to pandemic-led social-distancing measures and closure of public venues may present another less favorable environmental factor influencing young children’s vision health. In this regard, prior research has found outdoor playtime was associated with better uncorrected visual acuity [7], especially because outdoor lighting is categorically less fluorescent than indoors [8].

Notwithstanding, the potential vision health risks propagated by new norms in instructional and learning arrangements amidst the ongoing COVID-19 pandemic will likely add to an already serious global youth vision crisis [9]. Importantly, early myopia (near-sightedness) onset and progression among young children are especially concerning, not only because of its widespread prevalence and difficulty in proper mitigation applications [10] but also due to critically associated risks of lasting vision impairment [11]. In particular, excessive axial elongation of the eye associated with early myopia increases later-life risks of vision disease complications including macular degeneration, posterior staphyloma, retinal detachment, cataract, and glaucoma that could lead to blindness [12]. More worryingly, myopia onset is becoming increasingly prevalent among young children, particularly for girls in higher grades and in urban areas [13,14,15]. Studies have also shown that the earlier children become myopic, the more likely they are to develop high myopia, and the worse the prognosis [16].

From a physiological perspective, in vivo studies employing infant monkeys have suggested that extended relative peripheral hyperopic defocus stimulation can alter central refractive development [17], which could likely induce compensating axial myopic eye growth and premature refractive vision development [18]. For instance, it has been shown using inflammatory markers in mice that blue light emitted from computer screens has potentially harmful effects on the retinal pigment epithelium, which may result in axial elongation and development of pathological myopia [19]. In addition, outdoor eye use under natural light is commonly associated with increased depth of focus and reduced eye strain, which are inversely related to axial elongation [20]. In studies employing in vivo models, visual experiments have shown that poor lighting conditions can lead to excessive vitreous chamber lengthening, such that low-light level can result in axial elongation and refractive myopic excursions, while high-light level is associated with lower rates of form-deprivation myopia [21].

Critically, while existing studies have independently examined near-vision electronic use, outdoor activity, and lighting condition, the interrelated relationship among the three have not been assessed in conjunction, and their relative risks are unknown. In this study, we examined the association between digital screen use, outdoor activity, lighting condition, and myopia development among school-age children in China during the COVID-19 pandemic. 

## 2. Methods

### 2.1. Study Subjects

Schools in China were closed between January and May, 2020 due to the COVID-19 pandemic. An anonymous online survey was conducted to collect school-age children’s responses regarding their background information, time use, and vision condition during this period of pandemic-led school closure. The questionnaire was distributed from 12 to 18 May 2020 via a nationally known education press, which solicited respondents from 29 provinces and autonomous regions. Completing the questionnaire takes about 10–15 min online. The inclusion criteria for participants were as follows: (1) literate and can understand the questionnaire; (2) enrolled in primary, lower-secondary, or upper-secondary schools; (3) voluntary participation; (4) submitted only one response using the same IP address; (5) whose guardian has submitted informed consent. A total of 3405 respondents from 1st to 12th grade satisfied the study’s inclusion criteria. This study was approved by the Institutional Review Board of Shaanxi Normal University, and the study was conducted according to the World Medical Association Declaration of Helsinki.

### 2.2. Construct Measures

In order to facilitate the investigation, items on the questionnaire prompted respondents to self-evaluate symptomatic changes in their vision condition using the Lay Terms Approach, which advised using terminology that subjects are familiar with [22], such as “blurry vision when looking at distant objects” or “the need to squint.” The standardized questionnaire also collected information on time (hours) spent using e-learning devices such as TVs, computers, or smartphones, and how frequent subjects engaged in outdoor exercise, as well as their subjective ratings of indoor lighting condition (too dim, too bright, or feels okay) while using e-learning devices. Following previous studies, we classified e-learning device use into three categories by device type—near (0.1 m for smartphones), intermediate (0.5 m for computers), and far (0.8 m for TVs)—and calculated daily digital screen use in diopter hours (dh), which is a viewing-distance weighted measure of near-vision exposure to digital devices [23].

dh = (3 × hours viewing at 0.1 m) + (2 × hours viewing at 0.5 m) + (1 × hours viewing at 0.8 m)

### 2.3. Statistical Analysis

In the first analytic step, we conducted univariate non-parametric analysis and reported chi-square test, as well as reporting parametric paired sample t-test results, in order to assess to what extent self-reported progression of myopic symptoms (dependent variable) differs by individual characteristics. A *p*-value of <0.05 was considered to be statistically significant. In the second analytic step, we fit multivariate logistic regression models to examine the association between digital screen use, outdoor activity, lighting condition, and self-reported myopia progression, after controlling for individual traits and pre-pandemic vision condition. The analyses were performed using STATA version 15.0 (Stata, StataCorp LLC, College Station, TX, USA) software.

## 3. Results

In Table 1, descriptive statistics of subject background information and results from the univariate nonparametric analysis are presented. Of the 3405 subjects that satisfied the inclusion criteria, 1358 (39.9%) reported myopic symptoms, 1647 (48.4%) were female, 2234 (65.6%) were in primary, 269 (7.9%) were in lower-secondary, and 902 (26.5%) were in upper-secondary schools, 540 (15.6%) were in rural areas, 248 (7.3%) were in urban–rural transitional areas, and 2627 (77.1%) were in urban cities. Among them, 1374 (40.4%) reported suffering from myopia prior to the COVID-19 pandemic. In terms of daily digital screen use, we first calculated daily digital screen use in diopter hours (dh), for which the sample mean is 10.0 diopter hours (SD = 6.3). We also reported daily digital screen use in unadjusted hours, which has a sample mean of 3.9 h (SD = 2.3). As for outdoor exercise, 620 (18.2%), 398 (11.7%), 1583 (46.5%), and 804 (23.6%) subjects reported as frequent, somewhat frequent, somewhat infrequent, and infrequent, respectively. For indoor lighting condition, 208 (6.1%) reported it being “too dim,” 96 (2.8%) “too bright,” and 3101 (91.1%) as “feels okay.”

Under univariate nonparametric analysis, we examined the association between subject background characteristics, daily digital screen use, outdoor exercise, indoor lighting condition, and progression of myopic symptoms during the COVID-19 pandemic. First, myopic symptoms do not differ by subjects’ sex (χ^2^ = 1.124, *p* = 0.289) or location of residence (χ^2^ = 3.452, *p* = 0.178). Second, subjects who reported myopic symptoms are more likely to be in lower-secondary and upper-secondary but less so in primary schools (χ^2^ = 179.580, *p* < 0.001). Third, subjects who suffer from a pre-pandemic myopia condition are also more likely to report symptomatic myopia progression during the COVID-19 pandemic (χ^2^ = 338.785, *p* < 0.001). Fourth, subjects who reported myopic symptoms on average engage in 3.7 more diopter hours than subjects who did not (*p* < 0.001). The same finding holds true without diopter adjustment, which is 1.4 unadjusted more so for subjects who reported myopic symptoms (*p* < 0.001). Fifth, subjects’ frequency of participation in outdoor exercise is associated with the likelihood of reporting myopic symptoms (χ^2^ = 36.015, *p* < 0.001). Sixth, indoor lighting condition, particularly lighting condition that is too dim or too bright, is associated with progression of myopic symptoms (χ^2^ = 68.347, *p* < 0.001).

**Figure 1:**
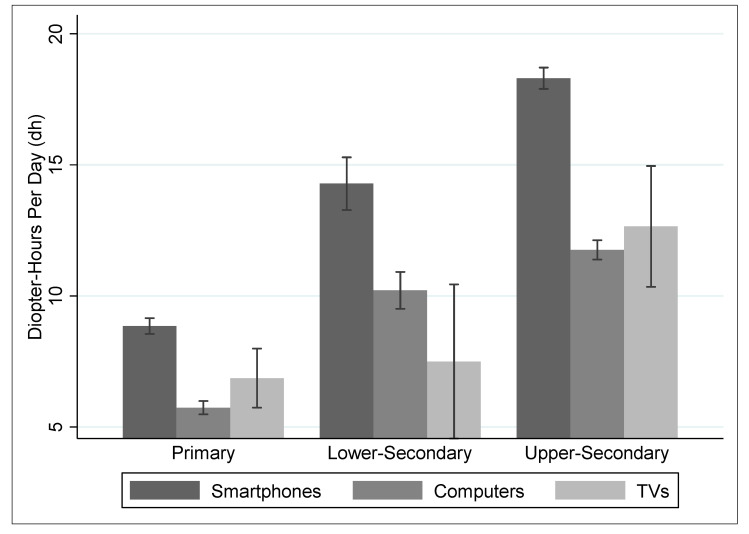
Daily use of e-learning devices among school-age children.

The distribution of the computed diopter hours result is displayed by enrolment level and by e-learning device type in Figure 1, from which two observations can be made. First, higher grade levels are associated with more intensive daily digital screen use in diopter hours. Second, smartphone is the most commonly used e-learning device reported across all grade levels.

The multivariate logistic regression analysis was used to examine the association between e-learning screen use, outdoor activity, lighting condition, and myopia development, after adjusting for subjects’ sex, grade, and location. Most strikingly, findings in Table 2 indicate that every diopter hour increase in daily e-learning screen use is significantly associated with progression of myopia symptoms (OR: 1.074, 95% CI: 1.058–1.089; *p* < 0.001). Since the sample average of daily digital screen use is 10.0 diopter hours, this result would imply substantial risks of myopic progression for the typical subject. In addition, subjects who engage in four to six times (OR: 0.745, 95% CI: 0.568–0.977; *p* = 0.034) and one to three times (OR: 0.829, 95% CI: 0.686–0.991; *p* = 0.048) of outdoor exercise per week are significantly less likely to report myopia symptoms than subjects who have no outdoor exercise each week. Finally, indoor lighting that is “too dim” (OR: 1.686, 95% CI: 1.226–2.319; *p* = 0.001) or “too bright” (OR: 1.529, 95% CI: 1.007–2.366; *p* = 0.036) is significantly associated with a higher likelihood of myopia symptoms, relative to the comfortable indoor lighting condition. The association between pre-pandemic myopia condition and progression of myopia symptoms is also statistically significant (OR: 2.814, 95% CI: 2.376–3.334; *p* < 0.001). 

## 4. Discussion

A substantial portion of primary and secondary schools in China were closed between January and May of 2020, and a majority of school-age children had to resort to online e-learning using computers, smartphones, or TVs. Importantly, these remote learning arrangements may present new risk factors for youth vision development as a consequence of changes in daily physical and eye-use behavior among children. To the best of our knowledge, this study is one of the first to examine the association between digital screen use, outdoor activity, lighting condition, and myopia development among school-age children in the context of a nationwide remote learning experiment during the COVID-19 outbreak in China.

Using a large-scale national survey, we presented three main findings. First, we found that the duration of daily digital screen use among school-age children during the COVID-19 outbreak in China is substantial, measuring at 3.9 h daily on average. Once weighting by viewing-distance is considered, near-vision exposure to digital devices rises to 10.0 diopter hours daily. Using multivariate logistic regression analysis, we found that each additional diopter hour of digital screen use is associated with a higher likelihood of symptomatic myopia development, which can translate into significant risks considering the extended periods of time young children spend in front of digital screens daily. Second, approximately one in four school-age children in our sample do not perform any outdoor exercise during the COVID-19 school closures, and more than 70 percent engage in outdoor exercise less than three times per week. Prior studies have highlighted the positive influence outdoor playtime can have on visual acuity as well as the associated health risks the lackthereof [7]. In our analysis, we found that more frequent outdoor exercise is generally associated with a lower likelihood of myopia development. Third, approximately one in ten school-age children in our sample report that indoor lighting condition is either too dim or too bright at home. As the main venue for learning activities during the COVID-19 pandemic, indoor lighting conditions critically affect children’s vision development, and poor lighting conditions are associated with a higher likelihood of worsening vision status among children in the study sample.

We contribute to the current literature on youth public health and build on prior studies on adolescent health that examine the association between near-vision electronic use [24], outdoor activity [7], lighting condition [21], and myopic vision progression by assessing these risk factors in conjunction and leveraging an extended period of COVID-19-pandemic-induced remote learning. While this study does not directly assess how student learning is affected, prior studies have underscored the critical negative impact myopic vision can have on student learning, particularly if corrective vision interventions are not afforded [25]. In this regard, our findings tend to confirm speculative predictions of a myopia boom during the COVID-19 pandemic [3] and are consistent with a recent longitudinal study on Chinese youth that indicated the positive association between digital screen exposure and prevalence of myopia [26] while complementing a recent cross-sectional study that examined how home confinement can have adverse effects on youth vision health [27], with richer information on digital screen use and indoor lighting condition. Based on our findings, we speculate that extended periods of school closure due to public health crises, such as the COVID-19 pandemic, and consequent alternative learning arrangements at home can increase the risks of inducing a myopia boom among school-age children. However, mitigation strategies such as limiting near-vision digital screen use duration, increasing outdoor exercise frequency, and improving indoor lighting conditions may prove to be effective in reducing, or delaying, such a youth visual health crisis [28]. While these findings are context-specific to school-age youth in China, the broader behavioral and policy implications are broadly relevant for a wide range of countries whose education system is making or is expecting to implement similar e-learning accommodations during the COVID-19 pandemic.

Finally, a limitation in this study worth mentioning is relying on subjects’ self-reports rather than specialist eye examinations. While professional ophthalmic evaluations would be ideal to obtain detailed information on refractive error, prior studies have suggested that subjects’ self-assessment of vision status does not differ systemically from professional ophthalmic evaluations [29]. Additionally, another reason for adopting self-reported measures is to allow for rapid and large-scale survey rollout [30], which would not have been feasible given the logistical and social-distancing requirements due to the COVID-19 pandemic. Nonetheless, it has been increasingly common in optometry and visual science studies to leverage questionnaire survey designs, considering the relatively high cost-effectiveness [24]. Future studies may find it useful to conduct professional ophthalmic evaluations in lieu of collecting subject self-reports.

## 5. Conclusions

The public health consequences stemming from a pandemic can be both wide-ranging and long-lasting, affecting not only the most vulnerable, but also leave its mark on the next generation in profound ways. In this study, we identified the less-visible vision health concerns on young children as many education systems have transitioned to remote online instruction due to the COVID-19 pandemic. Most strikingly, near-vision e-learning device use is a critical source affecting myopia development, and the associated youth health risks, as well as appropriate mitigation strategies, need to be seriously considered, should remote learning programs continue due to prolonged future waves of the COVID-19 pandemic.

## Figures and Tables

**Table 1:** Univariate parametric and nonparametric analysis.

Variable		Total (%)		Progression of Myopic Symptoms (Yes = 1, No = 0)				
				n		%		*p*
**Progression of Myopic Symptoms**								
Yes	39.9		-		-		-	
No	60.1		-		-		-	
**Sex ^a^**								
Female	48.4		672		40.8		0.289	
Male	51.6		686		39.0			
**Grade of Enrolment ^a^**								
Primary	65.6		711		31.8		0.000	
Lower-Secondary	7.9		135		50.2			
Upper-Secondary	26.5		512		56.8			
**Location of Residence ^a^**								
Rural	15.6		196		37.0		0.178	
Urban–Rural	7.3		92		37.1			
Urban	77.1		1070		40.7			
**Pre-Pandemic Myopia Condition ^a^**								
Yes	40.4		806		58.7		0.000	
No	59.6		552		27.2			
e-Learning Screen Use, diopter hours per day (mean, s.d., range) ^b^		10.0, 6.3, 2–21		mean (1) − mean (0) = 3.7				0.000
e-Learning Screen Use, unadjusted hours per day (mean, s.d., range) ^b^		3.9, 2.3, 1–10		mean (1) − mean (0) = 1.4				0.000
**Outdoor Exercise ^a^**								
Frequent (daily)	18.2		250		40.3		0.000	
Somewhat Frequent (4–6 times/week)	11.7		132		33.2			
Somewhat Infrequent (1–3 times/week)	46.5		588		37.2			
Infrequent (0 times/week)	23.6		388		48.3			
**Indoor Lighting Condition ^a^**								
Too Dim	6.1		133		63.9		0.000	
Too Bright	2.8		55		57.3			
Feels Okay	91.1		1170		37.7			

Notes: ^a^ *p*-value based on χ^2^ test, ^b^ *p*-value based on *t*-test.

**Table 2:** Multivariate logistic regression analysis.

Variables	Progression of Myopic Symptoms (Yes = 1, No = 0)		
	OR	95% CI	*p*
e-Learning Screen Use, diopter hours per day (dh)	1.074	1.058–1.089	0.000
**Outdoor Exercise**			
Frequent (daily)	0.994	0.788–1.255	0.962
Somewhat Frequent (4–6 times/week)	0.745	0.568–0.977	0.034
Somewhat Infrequent (1–3 times/week)	0.829	0.686–0.991	0.048
Infrequent (0 times/week)	1		
**Indoor Lighting Condition**			
Too Dim	1.686	1.226–2.319	0.001
Too Bright	1.529	1.007–2.366	0.036
Feels Okay	1		
**Sex**			
Female	0.990	0.853–1.149	0.895
Male	1		
**Grade of Enrolment**			
Primary	1.006	0.756–1.340	0.966
Lower-Secondary	0.907	0.676–1.217	0.514
Upper-Secondary	1		
**Location of Residence**			
Rural	0.988	0.801–1.220	0.913
Urban–Rural	0.872	0.651–1.167	0.514
Urban	1		
**Pre-Pandemic Myopia Condition**			
Yes	2.814	2.376–3.334	0.000
No	1		

Notes: OR = odds ratio; CI = confidence interval.

## Data Availability

Restrictions apply to the availability of data used, due to study subject privacy protection. Data was obtained from Teachers Daily and is available upon request with the permission of Teachers Daily.
